# Recent total synthesis of natural products leveraging a strategy of enamide cyclization

**DOI:** 10.3762/bjoc.21.81

**Published:** 2025-05-22

**Authors:** Chun-Yu Mi, Jia-Yuan Zhai, Xiao-Ming Zhang

**Affiliations:** 1 State Key Laboratory of Natural Product Chemistry & College of Chemistry and Chemical Engineering, Lanzhou University, Lanzhou 730000, P. R. China

**Keywords:** alkaloid, cyclization, enamide, natural product, total synthesis

## Abstract

Enamides are distinctive amphiphilic synthons that can be strategically incorporated into cyclization reactions. The iminium species generated from enamides via nucleophilic addition or substitution are capable of engaging in further electrophilic additions or isomerization processes. Exploiting the multiple reactivities of enamides facilitates the development of diverse cyclization modes that provide entries to various *N*-heterocycles, some of which serve as key structural motifs in natural alkaloids. This review highlights recent advancements in enamide-based cyclization reactions, including enamide–alkyne cycloisomerization, [3 + 2] annulation, and polycyclization, with a particular emphasis on their pivotal role as a strategy in the total synthesis of natural products.

## Introduction

The use of enamines as surrogates for enols in nucleophilic reactions has been well-documented for decades since their first report by Stork in the 1950s [1–3]. Compared with enols, enamines benefit from the lone pair of electrons on the nitrogen atom, which enhances the nucleophilicity of the alkene, enabling it to react with a broad range of electrophiles. This activation mode of carbonyl compounds has been so well-established that it is featured in nearly every organic chemistry textbook. However, despite their versatility, enamines themselves are not easily handling compounds in experimental settings. Their sensitivity to hydrolysis complicates their isolation and identification, and following the nucleophilic addition or substitution, the resulting iminium ions often undergo direct hydrolysis, preventing further use in a cascade nucleophilic addition. As a result, enamines are not ideal partners in tandem reactions for the synthesis of nitrogen-containing products. As analogues to enamines, the enamides contain an *N*-acyl group in place of the original alkyl group. The electron-withdrawing effect of the amide group delocalizes the nitrogen lone pair, thereby reducing the electron density and nucleophilicity of the enamide double bond. These features significantly diminish the reactivity of enamides as nucleophiles, rendering them more stable than enamines. This stability is reflected in their frequent occurrence in natural products [4]. As a result, research on the synthetic applications of enamides has historically lagged behind that of enamines [5–6]. Beyond their use in hydrogenation reactions [7–8], the exploration of enamides’ nucleophilic reactivity has only gained momentum in recent years. Inspired by pioneering work from various research groups [9–15], the potential of enamides in nucleophilic reactions has become recognized. Among them, enamide cyclizations have attracted considerable attention due to their promise in the total synthesis of alkaloids [16]. Notably, these valuable compounds can be employed as efficient synthons in enamide–alkyne cycloisomerization, [*n* + *m*] cycloadditions, pericyclic reactions, and radical cyclizations. A comprehensive review of these advancements up until 2015 has already been documented [16]. In this review, recent breakthroughs of these enamide cyclizations will be surveyed from the viewpoint of natural product synthesis. Leveraging the enamide–alkyne cycloisomerization cyclizations, *Lycopodium* alkaloids (−)-dihydrolycopodine, (−)-lycopodine, (+)-lycoposerramine Q, (+)-fawcettidine, (+)-fawcettimine, and (−)-phlegmariurine have been synthesized in a concise and efficient manner, while employment of the [2 + 3] cycloadditions or a polycyclization enables the elegant total synthesis of *Cephalotaxus* alkaloids cephalotaxine, cephalezomine H, (−)-cephalotaxine, (−)-cephalotine B, (−)-fortuneicyclidin A, (−)-fortuneicyclidin B, and (−)-cephalocyclidin A.

**Figure 1:**
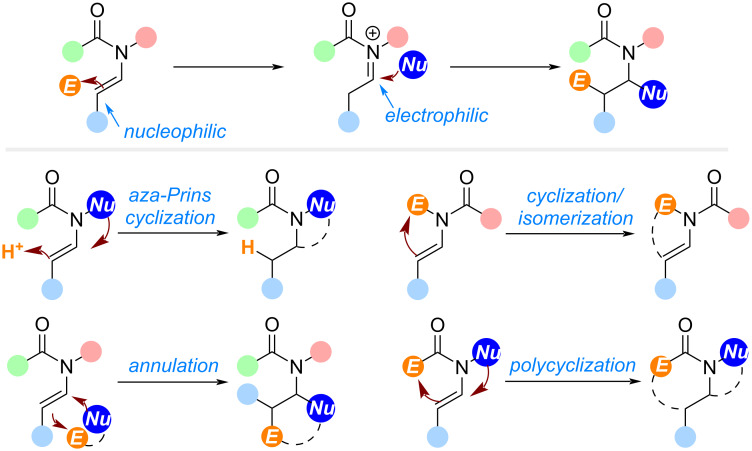
Reactivity of enamides and enamide cyclizations.

Unlike enamines, tertiary enamides can participate in cyclization reactions initial as nucleophiles, and upon protonation, alkenylation, or alkylation, the resultant iminium intermediates can serve as electrophiles. Due to the presence of an amide, the resulting iminiums from the enamides can be stabilized to take part in the second nucleophilic addition, though direct isomerization of the iminiums to the enamides is also possible (Figure 1). Guided by these principles, tandem reactions or annulations can be designed to efficiently access *N*-heterocycles. As the enamides are also easily accessible via condensations, applications of these nitrogen-containing building blocks in the synthesis of *N*-heterocycles are synthetically straightforward. When applied properly, these methods offer promising strategies for the total synthesis of complex natural products.

## Review

### Aza-Prins cyclization – total synthesis of (−)-dihydrolycopodine and (−)-lycopodine

**Scheme 1:**
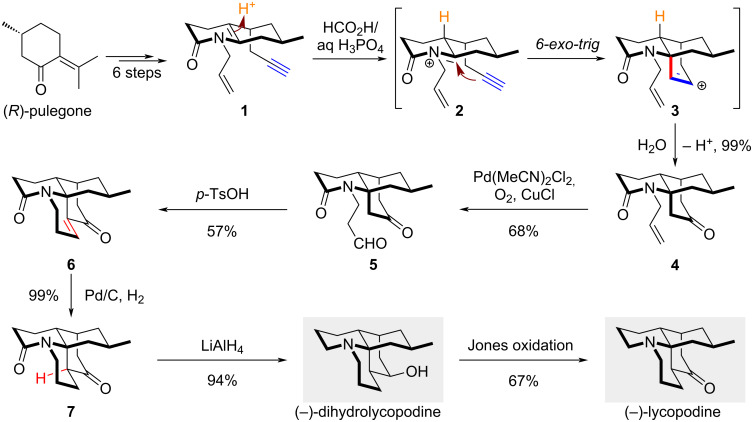
Total synthesis of (−)-dihydrolycopodine and (−)-lycopodine.

Cyclizations of enamides can proceed via several distinct pathways. If protonation of the enamide occurs first, the resulting iminium ion can be readily captured by a wide variety of nucleophiles, including alkenes and alkynes. These *aza*-Prins cyclizations have potential applications in the synthesis of natural alkaloids, as exemplified by She’s total synthesis of (−)-dihydrolycopodine and (−)-lycopodine [17]. These *Lycopodium* alkaloids have long been valued in traditional Chinese medicine for their therapeutic effects on skin disorders and as analgesics [18]. Preliminary biological evaluations also suggest their antipyretic and anticholinesterase activities [19]. As a prominent member of the lycopodine-type alkaloids, lycopodine features a characteristic tetracyclic structure with a bridged cyclohexanone. To address the challenges associated with constructing the complex ring systems of this structure, She and co-workers devised an intramolecular *aza*-Prins cyclization strategy to form both the bridge ring and the *N*-hetero quaternary center in a single step. As depicted in Scheme 1, key enamide **1** was prepared from (*R*)-pulegone in 6 steps. In the presence of the weak acid H_3_PO_4_, protonation of **1** generates a stabilized iminium ion **2**, which then undergoes a *6-exo-trig* cyclization to deliver **4** after hydration of cation **3**. Notably, the terminal alkene remains intact during this process, and the initial protonation proceeds with full stereocontrol, rendering this transformation both highly chemo- and diastereoselective. From the cyclization result, it is presumed that the higher nucleophilicity of the alkyne functionality over the terminal alkene and the conformational strain of forming a bridge[3.2.1]bicycle might be responsible for a selective *6-exo-trig* cyclization.

From tricyclic compound **4**, *anti*-Markovnikov oxidation catalyzed by palladium led to the formation of aldehyde **5**. When treated with *p*-TsOH, the intramolecular aldol condensation of **5** provided the tetracyclic α,β-unsaturated enone **6** in 57% yield. Subsequent catalytic hydrogenation using Pd/C conditions delivered the hydrogen to the alkene from the less hindered face, producing ketone **7** with high diastereoselectivity. Final reduction of both the amide and ketone groups completed the total synthesis of (−)-dihydrolycopodine, which could then be further oxidized to (−)-lycopodine. The entire synthetic route hinges upon the development of a sterically congested *aza*-Prins cyclization, enabled by the presence of the enamide and its neighboring alkyne. Building on this strategy, the authors also accomplished the total synthesis of (−)-lycospidine A in only 10 steps [20], another *Lycopodium* alkaloid with a truncated tetracyclic skeleton and distinct oxidation levels, further highlighting the versatility and efficiency of the enamide *aza*-Prins approach.

### Cyclization/isomerization – collective total synthesis of fawcettimine-type alkaloids

**Scheme 2:**
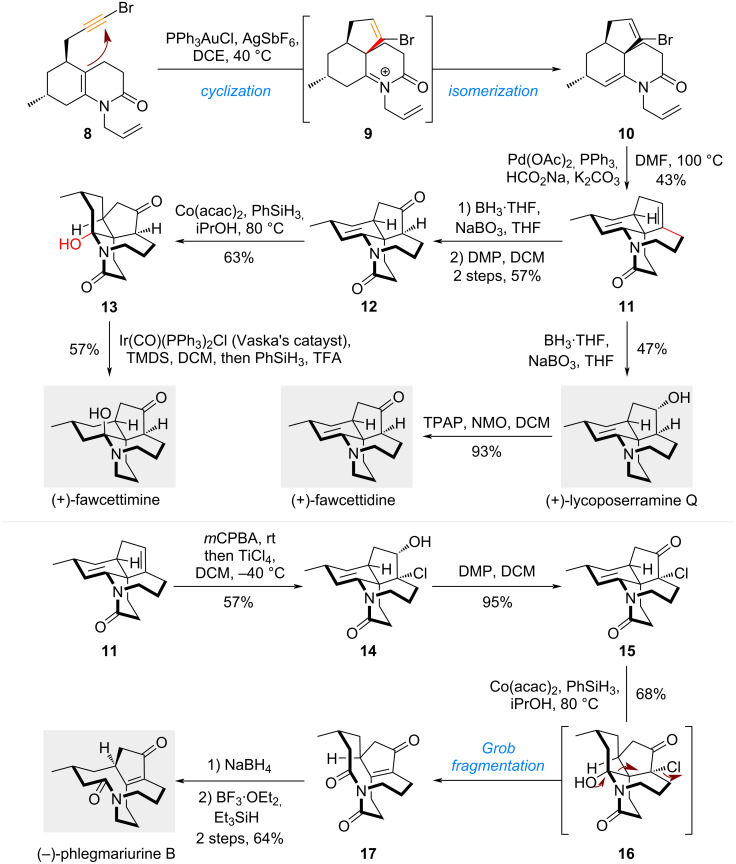
Collective total synthesis of fawcettimine-type alkaloids.

The bicyclic decahydroquinoline enamide motif can serve as a versatile precursor to access different types of tricyclic *N*-heterocycles. As demonstrated in the above work from She’s group, the *aza*-Prins cyclization renders the α-position of enamide to be an active cyclization site, with the alkyne tether acting as the nucleophile. Since it is well-established that alkynes, when activated by transition metals such as gold or platinum, can also function as electrophiles, modulating the reactivity of the decahydroquinoline enamide motif to enable an enamide–alkyne cycloisomerization is also feasible. In this case, the initial nucleophilic cyclization of the enamide is followed by isomerization, shifting the cyclization site from the α- to the β-position of the enamide, resulting in the formation of a fused triangular ring system rather than a bridged tricycle. Building on this strategy, the same research group developed a divergent synthetic route that culminated in the concise and collective total synthesis of a series of fawcettimine-type *Lycopodium* alkaloids (Scheme 2) [21], which are well-known for their potent acetylcholinesterase (AChE) inhibitory activities [18].

In the presence of a catalytic amount of PPh_3_AuCl and AgSbF_6_, the enamide–alkyne cycloisomerization of bromo-substituted alkyne **8** proceeded via a *5-endo-dig* cyclization to afford tricyclic compound **10** through the formation of iminium intermediate **9**. The azepane ring was then constructed via an intramolecular reductive Heck reaction from vinyl bromide **10** with exclusive regioselectivity. Considering the strain of forming the 7-membered ring, this highly efficient *7-endo-trig* (vs *6-exo-trig*) transannular Heck cyclization reaction was remarkable to be realized in a regioselective manner. From tetracyclic compound **11**, a one-pot facial and regioselective hydroboration/amide reduction followed by oxidation produced (+)-lycoposerramine Q, which was then converted to (+)-fawcettidine by Ley oxidation. Alternatively, hydroboration of **11** in mild conditions without the reduction of amide-generated ketone **12** after a subsequent Dess–Martin oxidation. Upon treatment of **12** with Co(acac)_2_ and PhSiH_3_ in iPrOH at 80 °C, the Mukaiyama hydration of enamide delivered hemiaminal **13**. Despite the incorrect configuration of the newly formed hydroxy group, it is considered inconsequential due to the reversibility of hemiaminal. Consequently, further reduction of the amide could complete the total synthesis of (+)-fawcettimine with in situ adjustment of the hemiaminal configuration.

The incorrect configuration observed in the Mukaiyama hydration also inspired the authors to develop a fragmentation process for the total synthesis of (−)-phlegmariurine B. A one-pot epoxidation/nucleophilic epoxide opening introduced both a hydroxy group and a chloride across the cyclopentene, producing **14** in 57% yield. After oxidation of alcohol **14** to ketone **15**, the Mukaiyama hydration then triggered a Grob fragmentation process of hemiaminal **16** and afforded the imide compound **17**. Final regioselective reduction of one of the two carbonyls on the imide completed the synthesis of (−)-phlegmariurine B.

### Annulation

#### Total syntheses of cephalotaxine and cephalezomine H

**Scheme 3:**
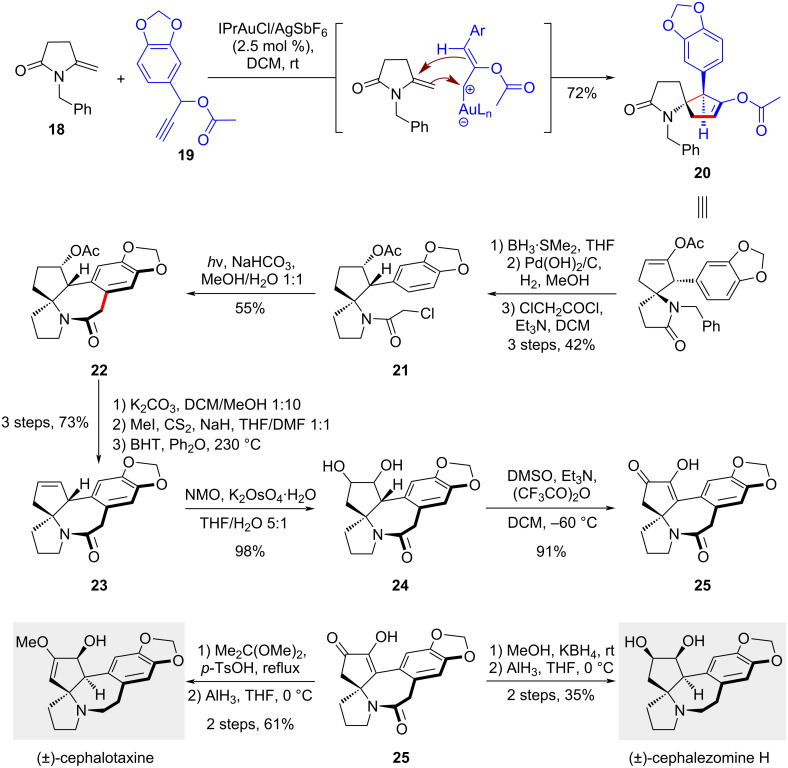
Total syntheses of cephalotaxine and cephalezomine H.

The [2 + 3] annulation of enamides is a relatively underexplored reaction, particularly in the context of total synthesis. Its synthetic potential remains to be fully excavated, as it offers a modular approach for disassembling molecules into segments of comparable sizes. Recently, Fan's group reported the development of this annulation and applied it in the divergent total synthesis of *Cephalotaxus* alkaloids (Scheme 3) [22], including cephalotaxine whose ester, homoharringtonine, has been listed as an approved FDA drug for the treatment of chronic myeloid leukemia [23]. In their elegant study, an Au-catalyzed [2 + 3] annulation was utilized to transform enamine **18** and propargyl ester **19** into 1-azaspiro[4.4]nonane **20** with high diastereoselectivity. Notably, the combination of an *N*-heterocyclic carbene gold catalyst and a silver salt AgSbF_6_ was found to be essential in guaranteeing the reactivity of the alkyne partner, probably due to the formation of a more acidic cationic gold complex. Following this annulation, reduction of the amide in **20**, catalytic hydrogenation of the alkene and the *N*-benzyl group, and subsequent nitrogen acylation yielded chloride **21** in a 42% total yield, setting the stage for the Witkop photocyclization. This transformation was carried out using a high-pressure mercury vapor lamp to afford benzazepine **22**, completing the construction of the pentacyclic framework of the natural product. Subsequent functional group manipulations, including the Chugaev elimination of the hydroxy group on the cyclopentane ring, dihydroxylation, and oxidation of the diol to a diketone, produced intermediate **25** in its enol form. From this common intermediate, regioselective etherification at the less hindered position formed an enol ether. Final reduction of both the amide and the ketone using alane completed the total synthesis of cephalotaxine. Similarly, diastereoselective reduction of **25** with KBH_4_ followed by alane reduction provided another alkaloid cephalezomine H.

#### Collective total syntheses of *Cephalotaxus* alkaloids

**Scheme 4:**
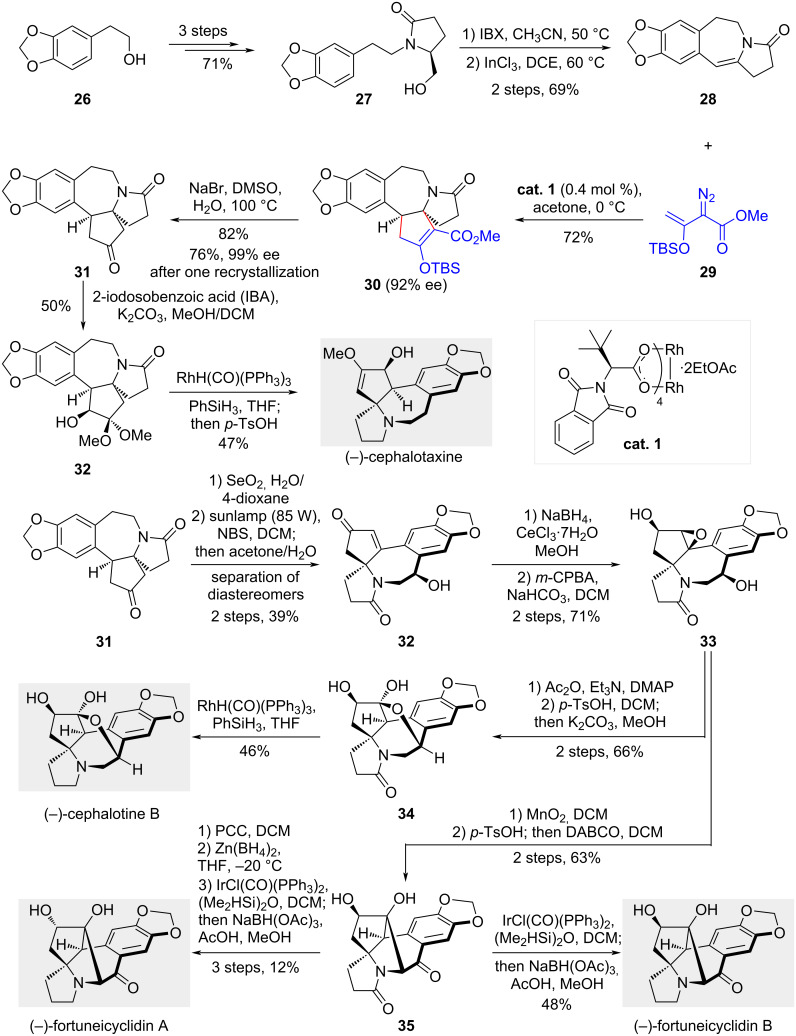
Collective total syntheses of *Cephalotaxus* alkaloids.

The cyclopentane ring in most *Cephalotaxus* alkaloids is characterized by the highest oxidation state within the pentacyclic framework. Installation of this cycle with suitable functional handles usually forms the key strategy of numerous total syntheses of these alkaloids [24–26]. Building upon earlier work, the same research group further advanced this approach by developing a Rh-catalyzed asymmetric [2 + 3] annulation of tertiary enamides with enoldiazoacetates, enabling highly efficient total syntheses of *Cephalotaxus* alkaloids (Scheme 4) [27]. In their recent study, the homopiperonyl alcohol **26** was transformed into tricyclic enamide **28** in five steps in a decagram scale. As no column chromatography was required during this process, the synthetic route is highly practical. The enantioselective annulation of tertiary enamide **28** with enoldiazoacetate **29** was then explored under the catalysis of a chiral dirhodium catalyst. While Doyle and co-workers had previously reported an elegant [2 + 3] cycloaddition of secondary enecarbamates [28], the extension of this reaction to enamides lacking an N–H group is a notable advancement. After extensive optimization, the chiral dirhodium catalyst **cat. 1** was found to be most capable in terms of both stereocontrol and efficiency. The use of 0.4% amount of **cat. 1** provided adduct **30** in 72% yield with 92% enantioselectivity, and the reaction could be scaled up to decagrams. Subsequent decarboxylation and recrystallization of the resulting ketone **31** yielded an enantiopure product (99% ee), which serves as a versatile intermediate for the divergent total synthesis of several *Cephalotaxus* alkaloids.

The α-hydroxylation of cyclopentanone, followed by amide reduction and methanol elimination in one-pot, produced (−)-cephalotaxine in 9 steps. Alternatively, Riley SeO_2_ oxidation of **31**, benzylic bromination/hydrolyzation, facial selective ketone reduction, and epoxidation delivered compound **33** with the required oxidation level of the cyclopentane ring. In the final stages, Meinwald rearrangement/hemiketalization in a step-wise procedure, followed by amide reduction, completed the total synthesis of (−)-cephalotine B. Alternatively, after benzylic oxidation, the Meinwald rearrangement/aldol reaction gave rise to the bridge cyclic intermediate **35**, which can finally be converted into both (−)-fortuneicyclidin A and (−)-fortuneicyclidin B.

### Polycyclization

#### Cyclization/Pictet–Spengler reaction

**Scheme 5:**
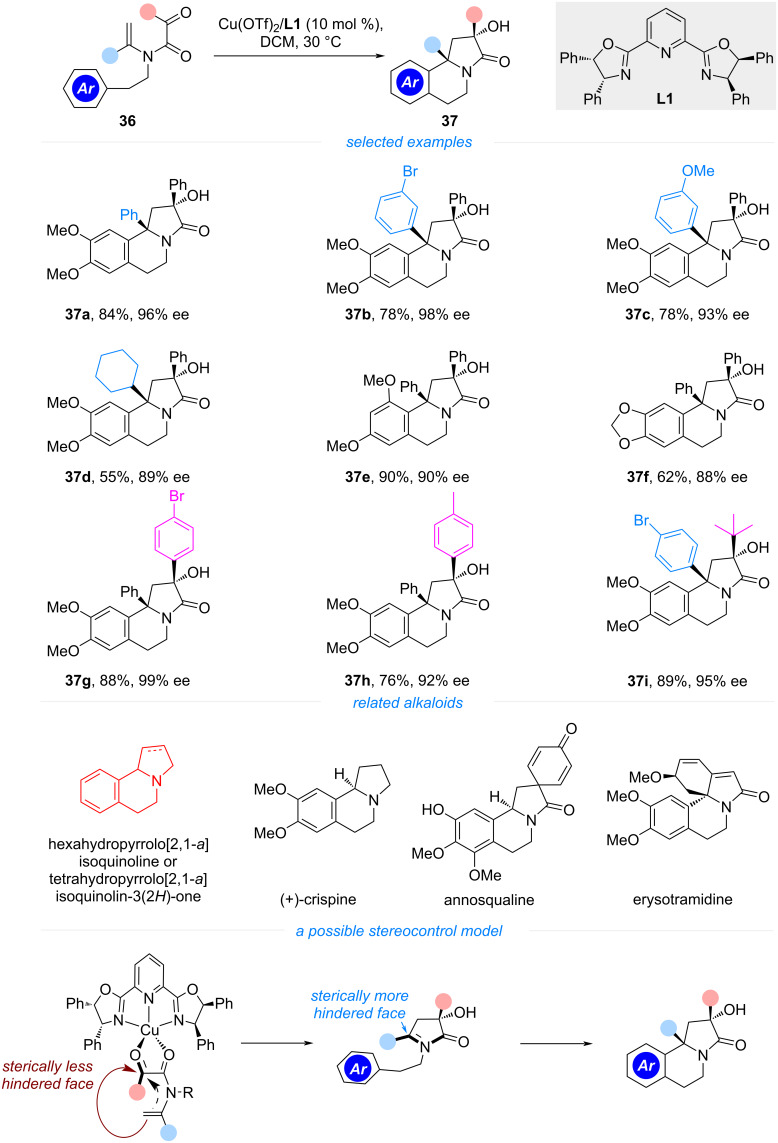
Asymmetric tandem cyclization/Pictet–Spengler reaction of tertiary enamides.

The hexahydropyrrolo[2,1-*a*]isoquinoline or tetrahydropyrrolo[2,1-*a*]isoquinolin-3(*2H*)-one framework is a pivotal core structure among various pyrrolo[2,1-*a*]isoquinoline alkaloids, exemplified by (+)-crispine, annosqualine, and erysotramidine, among others (Scheme 5) [29]. These bioactive alkaloids exhibit a broad spectrum of biological activities, including antitumor, antibacterial, antiviral, and antioxidizing properties. Previous synthetic strategies for these molecules typically rely on multi-step procedures to assemble the tricyclic core. However, the direct catalytic enantioselective formation of this scaffold from a linear precursor remains underexplored, despite the potential for such a tandem reaction to provide a more efficient route to these complex structures. In 2016, Wang and co-workers indigenously designed and developed a cyclization/Pictet–Spengler reaction cascade, leveraging the nucleophilicity of the tertiary enamides and the electrophilicity of the resulting acyliminium [30]. Unlike the monocyclization, which involves deprotonation of the acyliminium ion, the success of this polycyclization relies on the interception of the acyliminium ion by an aryl nucleophile, resulting in the formation of *N*-heterocyclic fused[6,6,5]tricycles. Optimization studies identified the tetraphenyl-substituted PyBox ligand **L1** as particularly effective in controlling the stereochemistry of the polycyclization, yielding high enantioselectivity for most substrates. As illustrated in Scheme 5, tertiary enamides with a tethered electron-rich arene could undergo cyclization to form products in high yields and excellent enantioselectivities. Notably, only a single diastereomer was produced in each case. The single-crystal X-ray crystallography revealed a *cis*-configuration for both the alkene and ketone substituents on the enamide, indicating that the intramolecular attack of the electron-rich arene on the acyliminium ion occurs from the *Si*-face. This stereochemical outcome is attributed to the steric discrepancy of the phenyl or *tert*-butyl group and the hydroxy group. The resulting tricyclic products could be further elaborated by elimination or amide reduction to yield hexahydropyrrolo[2,1-*a*]isoquinoline or tetrahydropyrrolo[2,1-*a*]isoquinolin-3(2*H*)-one frameworks, characteristic of alkaloids such as crispine analogs and erysotramidine.

**Scheme 6:**
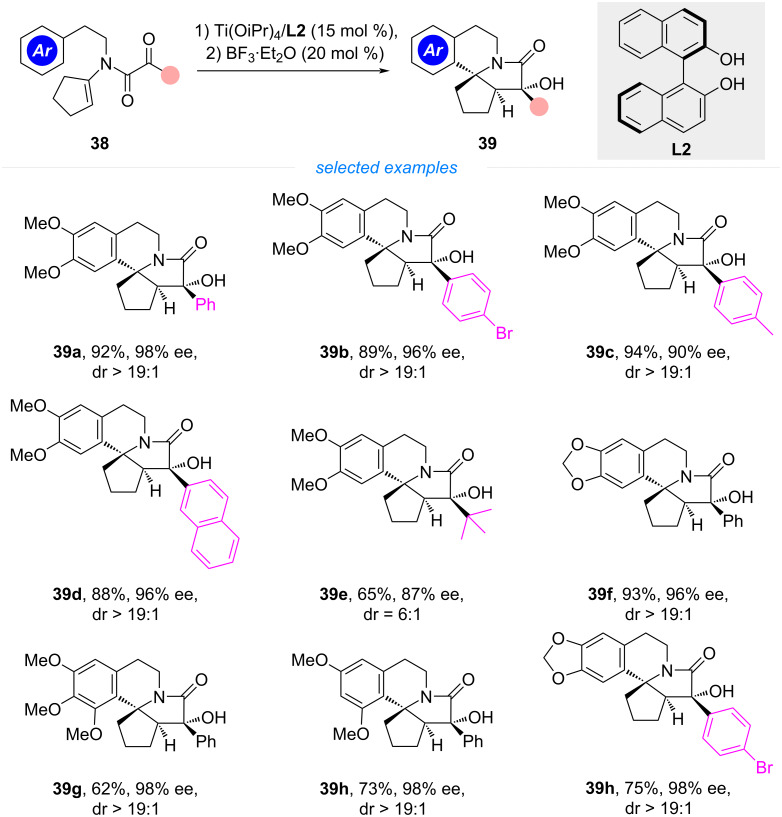
Tandem cyclization/Pictet–Spengler reaction for the synthesis of chiral tetracyclic compounds.

Building on their previous work on cyclization/Pictet–Spengler reaction, the same group further designed cyclopentanone derived tertiary enamides as cyclization precursors (Scheme 6). The analogous polycyclization generated a tetracyclic *N*-heterocycle with three continuous stereogenic centers, one of them being an *aza*-quaternary carbon [31]. The resulting fused ring-system structurally resembles the nucleus of erysotramidine alkaloids, though it features a truncated cyclopentane rather than the characteristic cyclohexane or cyclohexene. In their optimization studies, the authors found the sequential catalysis of a chiral binol–Ti complex and BF_3_·Et_2_O to be the most efficient, providing products **39** in high yields with excellent diastereo- and enantioselectivities. The substituent on the enamide could be varied from aryl to *tert*-butyl groups, though the terminating aryl group still necessitates an electron-rich arene. As was found in their previous work, the steric hindrance of the phenyl or *tert*-butyl group was supposed to be responsible for the excellent diastereoselectivity observed in the second cyclization process. In their later studies, the authors also found cyclohexanone-derived tertiary enamides to be viable substrates for the polycyclization [32], affording erythrinane core skeletons in high yields. However, in these cases, the use of a chiral Cr(III)(salen)Cl complex in combination with InCl_3_ was necessary to maintain a high level of stereocontrol.

#### Total synthesis of (−)-cephalocyclidin A

**Scheme 7:**
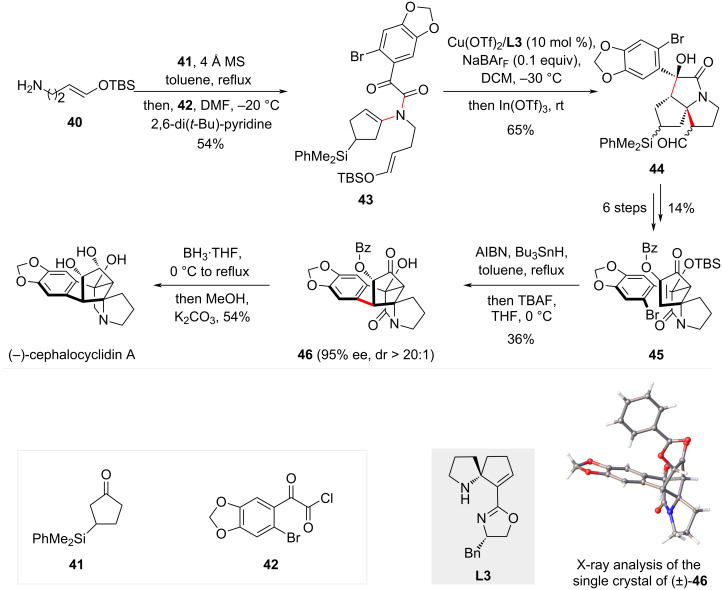
Total synthesis of (−)-cephalocyclidin A.

The bicyclic and tricyclic *N*-heterocycles without a fused arene are essential core structures in a wide array of alkaloids. A notable example is (−)-cephalocyclidin A, a cytotoxic pentacyclic cephalotaxus alkaloid [33–34]. Although the molecular structure contains a benzo-bridge ring system, disconnection of this bridge reveals a critical tricyclic *N*-heterocycle. To efficiently synthesize this tricycle, polycyclization of tertiary enamides is employed. Inspired by Wang’s earlier work, Zhang and Tu’s group developed a tandem cyclization/Mannich reaction to construct this architecture [35]. However, unlike the electron-rich arenes, the use of silyl enol ethers to terminate the second cyclization of the acyliminium intermediate would meet challenges associated with the instability of enolate derivatives. In their recent study, they successfully developed such a polycyclization taking advantage of a novel spiropyrroline-derived oxazole (SPDO) ligand (**L3**). As shown in Scheme 7, one-pot condensation of primary amine **40**, β-silyl substituted cyclopentanone **41**, and acyl chloride **42** produced enamide **43**. The polycyclization then took place under the catalysis of Cu(OTf)_2_/**L3** and In(OTf)_3_, delivering tricyclic product **44** in high yield with excellent enantioselectivity. Despite formation of multiple diastereomers due to the presence of silyl and aldehyde groups on the tricycle, it is inconsequential as these groups are either removed or oxidized in subsequent steps. After adjustment of the oxidation levels, the cyclopentenone **45** obtained was subjected to an intramolecular Giese reaction, producing **46** with establishment of the bridge cycle [36–37]. The excellent diastereoselectivity in this radical cyclization was further rationalized by DFT calculations, which suggests an energy discrepancy of the hydrogen atom transfer process from different faces of the resulting α-hydroxyl radical. Final reduction of the ketone and amide followed by deprotection completed the total synthesis, giving rise to (−)-cephalocyclidin A in 10 steps from known compounds.

## Conclusion

In summary, the perception of enamides as stable chemical entities with limited utilities in organic synthesis has evolved, and these compounds are now widely used in various cyclization reactions that play a pivotal role in the total synthesis of natural alkaloids. The nucleophilicity of enamides and the electrophilicity of the resulting acyliminium intermediates can be strategically manipulated in numerous ways to design cyclization and annulation reactions. Notably, these reactions – particularly tandem processes – are highly effective in constructing both fused and bridged ring systems, offering valuable new tools for chemical synthesis. Future advancements in the field could involve further applications of enamide cyclizations with other nucleophiles or in combination with other reaction patterns, potentially opening new avenues for the total synthesis of natural products.

## Data Availability

Data sharing is not applicable as no new data was generated or analyzed in this study.
